# Efficacy of Exercise Rehabilitation in Patients with Atrial Fibrillation after Radiofrequency Ablation: A Meta-Analysis of Randomized Controlled Trials

**DOI:** 10.1155/2022/9714252

**Published:** 2022-10-07

**Authors:** Yue Zhang, Pengna Ren, Ailing Tang, Li Dong, Xiaoyi Hu, Hong Wang, Fanglei Xu

**Affiliations:** ^1^Tongji University School of Medicine, Shanghai 200092, China; ^2^Department of Cardiology, Tongji Hospital Affiliated to Tongji University, Shanghai 200065, China; ^3^Department of Nursing, Tongji Hospital Affiliated to Tongji University, Shanghai 200065, China

## Abstract

**Background and Aims:**

Radiofrequency ablation is a commonly used treatment for paroxysmal atrial fibrillation (AF), but postoperative rehabilitation exercises are needed to reverse left ventricular structural and functional abnormalities. This meta-analysis aimed to evaluate the intervention effect of exercise training in patients with AF after radiofrequency ablation.

**Methods:**

A systematic literature search was conducted to identify articles in PubMed, MEDLINE, EMBASE, and the Cochrane Library from January 1, 2010 to December 1, 2021. The mean difference with 95% CI was pooled for continuous variables. We used Review Manager 5.3 for the standard meta-analysis. This study followed the guidelines of Preferred Reporting Items for Systematic Reviews and Meta-Analyses (PRISMA).

**Results:**

Ten randomized controlled trials (RCTs) were included, with a total of 892 patients with AF. The quality of one study was grade A, and the rest were grade B. The results of the meta-analysis showed that the improvement of 6 min walking distance (MD = 34.42, 95% CI: 3.20 to 65.63, *P*=0.03), peak oxygen uptake (MD = 1.96, 95% CI: 1.14 to 2.78, *P* < 0.001), left ventricular ejection fraction (LVEF) (MD = 0.09, 95% CI:0.01–0.17, *P*=0.02), resting heart rate (MD = −4.50, 95% CI: −8.85 to −0.14, *P*=0.04), and physical component summary (PCS) (MD = 3.00, 95% CI: 0.46 to 5.54, *P*=0.02) in the experimental group was significantly better than that of the control group, and the difference was statistically significant.

**Conclusion:**

Exercise training can improve the level of exercise endurance and cardiac function in patients. However, the results were limited by the quantity and quality of the studies. Large samples and high-quality studies are still needed to verify its long-term efficacy.

## 1. Introduction

Atrial fibrillation (AF) is the most common arrhythmia, and its morbidity and mortality show an increased tendency with age [1, 2]. It can cause heart failure, stroke, thromboembolism, and other serious life-threatening events [3]. According to a survey [4], there are about 8 million cases of AF in China, accounting for about 0.77%. AF has become an important health problem that affects global health and hinders social and economic development. The current treatments for AF include drug therapy and surgical treatment (radiofrequency ablation (RFA)). Left atrial appendage occlusion (LAAO) RFA has become the first choice for treating AF due to its minor trauma and fast postoperative recovery [5]. However, there is a recurrence rate of about 30% after catheter ablation [6]. Even after secondary ablation, the recurrence rate is as high as 7% to 24% [4], and postoperative intervention is still required to improve the structure and function of the heart.

Postoperative patient management focuses on controlling heart rate and rhythm, reducing the risk of stroke and related adverse cardiovascular events, and improving lifestyle. However, there is a lack of emphasis on routine exercise therapy after AF. Exercise rehabilitation is a comprehensive assessment of individual health, physical strength, cardiovascular function, exercise tests, and risk stratification. Doctors prescribe the corresponding exercise prescriptions to help patients recover physically and mentally. Several studies [7, 8] have confirmed that exercise rehabilitation plays a vital role in improving coronary heart disease, heart failure, myocardial infarction, and other cardiovascular diseases, but few studies report exercise rehabilitation used in AF patients after RFA. There are no systematic studies to clarify its role in the management of AF, and no guidelines or consensus have been formed. Previous studies on the impact of exercise rehabilitation on AF focused on the whole population of AF, and there was no meta-analysis after AF ablation. This study aimed to explore the improvement effect of exercise on patients with AF after RFA and to provide evidence for the formulation of postoperative rehabilitation training.

## 2. Materials and Methods

### 2.1. Search Strategy

A literature review was conducted in the Cochrane Central Register of Controlled Trials (CENTRAL), Cochrane Library Database of Abstracts of Reviews of Effects (DARE), MEDLINE Ovid, Embase Ovid, Web of Science, CINAHL EBSCO, PubMed, Scopus, CBM, China Knowledge Network, WanFang, and VIP databases to identify eligible publications in Chinese and English. The search period was from January 2010 to July 2021. The Chinese search keywords were “atrial fibrillation,” “exercise,” “aerobic exercise,” “resistance training,” “exercise training,” “exercise rehabilitation,” and “activity.” The English search keywords were “atrial fibrillation,” “aerobic exercise,” “exercise rehabilitation,” “training/exercise” and “randomized controlled trial.” We also manually searched the references of relevant reviews, systematic reviews, and included studies to identify other potentially eligible studies.

### 2.2. Inclusion and Exclusion Criteria

#### 2.2.1. Inclusion Criteria

The inclusion criteria were as follows:

(1) Study type: randomized controlled trials (RCTs). (2) Patients: adult patients 18 years of age or older who suffered from AF and received RFA, with informed consent obtained. (3) Interventions: the control group received routine care, including drugs, RFA, psychological counseling, health education, and discharge guidance; the experimental group implemented a structured exercise program on the basis of the corresponding control group, including aerobic, resistance exercise training, joint training (aerobic and resistance), functional electrical stimulation, and physical therapy of inspiratory muscle training. (4) Outcomes: 6-minute walking distance (6MWD), peak oxygen uptake (peak VO_2_), resting heart rate, left ventricular ejection fraction (LVEF), and quality of life.

#### 2.2.2. Exclusion Criteria

The exclusion criteria were as follows:

(1) The full text was not available; (2) relevant outcome indicators were not mentioned or unclearly expressed; (3) duplicate publications; and (4) animal studies, case reports, comments, abstracts, meeting minutes, and editorials.

### 2.3. Data Extraction and Quality Assessment

Two reviewers conducted the articles' screening processes and extracted the required information. If there were any disagreements, a third reviewer was consulted to make a final decision. The content of the information included: (1) authors and their country, publication year; (2) number of study participants, grouping; (3) intervention measures: intervention content, intervention frequency/period of each group; and (4) outcome indicators. The Cochrane risk-of-bias tool was used to evaluate the quality of RCTs, including random sequence generation, allocation concealment, blinding of participants and personnel, blinding of outcome assessment, incomplete outcome data, selectivity reporting, and other sources of bias [9]. If the included literature ultimately met the above items with low bias, its quality grade was A; if it partially conformed to these items with moderate bias, its quality grade was B; and if it was utterly noncompliant, its quality grade was C, and such a study should be excluded.

### 2.4. Data Analysis

The Review Manager (RevMan 5.3) software was used for the meta-analysis. The mean difference (MD) with 95% CI was pooled for continuous variables. The significance level was set at 0.05, with a 2-tailed test used. The *I*^2^ statistic was used to evaluate heterogeneity between studies, and a value of >50 indicated significant heterogeneity. Because of the small number of studies, we did not test publication bias because any test would have had low power to distinguish between chance and real asymmetry. We assessed the risk of bias in individual studies using the Cochrane Collaboration tool. The quality and consistency of the results were assessed by calculating the combined effects after excluding each selected study. We performed this meta-analysis in compliance with the guidelines set out in the Preferred Reporting Items for Systematic Reviews and Meta-Analyses (PRISMA).

## 3. Results

### 3.1. Search Results

**Figure 1:**
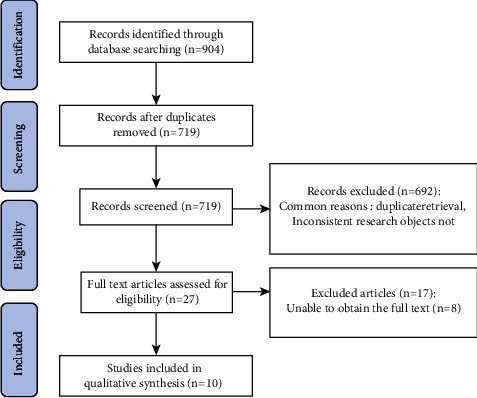
Flow chart of literature screening.

A total of 904 articles were retrieved. After removing 185 duplicate articles by NoteExpress software, ten RCTs were finally included [10–19], including eight in English [10–12, 15–19] and two in Chinese [13, 14]. The flow chart of literature screening is shown in Figure 1.

### 3.2. Basic Characteristics of the Included Studies

A total of 892 patients were included in this study, including 445 patients in the experimental group and 447 patients in the control group. The basic characteristics of the included studies are shown in Table 1.

### 3.3. Quality Assessment of the Included Studies

Among the collected literature, one study [12] was grade A, and the rest were grade B, with moderate overall quality. Eight studies [11–13, 15–19]described randomization methods; four studies [11, 12, 16, 17] described random allocation concealment; three studies [10, 12, 16] mentioned blinding methods, but only one [12] involved blinding of participants, personnel, and assessment; one study [16] had data loss. During the follow-up period, twenty-six patients in the experimental group and twenty-seven in the control group withdrew from the study (Table 2).

### 3.4. Meta-Analysis Results

#### 3.4.1. 6-Minute Walking Distance (6MWD)

**Figure 2:**
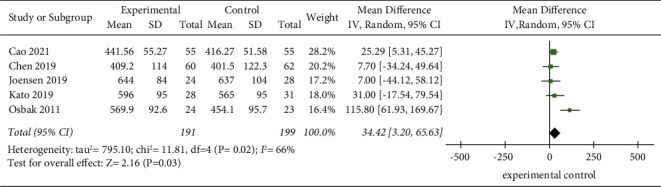
Meta-analysis of the comparison of the 6MWD between the two groups.

Five studies [10, 13, 14, 17, 18] evaluated the effect of exercise on the 6MWD of AF patients. Given that the trials pooled together are not drawn from the same population and also given the considerable clinical heterogeneity between the studies, only random-effects model should be used throughout the whole study and for all the analysis (*I*^2^ = 66%, *P*=0.02). The results showed that the 6MWD of the experimental group was significantly better than that of the control group (MD = 34.42, 95% CI: 3.20 to 65.63, *Z* = 2.16, *P*=0.03) (Figure 2). The sensitivity analysis showed that the study by Osbak et al. [10] was the cause of the heterogeneity of the results, but the analysis results did not change, indicating that the results were stable, as shown in Table 3.

#### 3.4.2. Peak Oxygen Uptake (Peak VO_2_)

**Figure 3:**
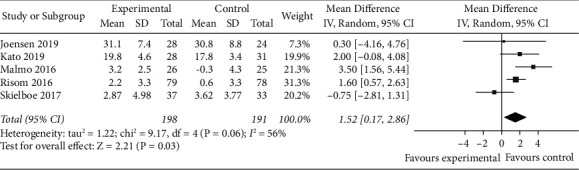
Meta-analysis of the comparison of peak VO_2_ between the two groups.

A total of five studies [15–19] reported the effect of exercise on peak VO_2_ of AF patients. The random-effects model was selected for meta-analysis (*P*=0.06, *I*^2^ = 56%). The results showed that the peak VO_2_ of the experimental group was significantly better than that of the control group, and the difference was statistically significant (MD = 1.52, 95% CI: 0.17 to 2.86, *Z* = 2.21, *P* < 0.05) (Figure 3). The sensitivity analysis showed that the study by Skielboe et al. [19] was responsible for the heterogeneity of the results, but there was no change in the analysis, indicating that the results were stable, as shown in Table 4.

#### 3.4.3. Resting Heart Rate

**Figure 4:**
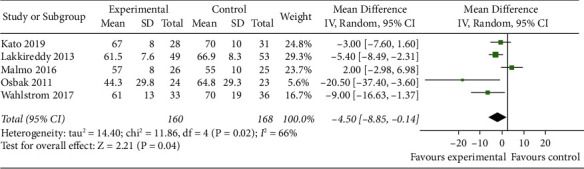
Meta-analysis of the comparison of resting heart rate between the two groups.

Five studies [10–12, 15, 17] evaluated the effect of exercise on the resting heart rate of AF patients. The heterogeneity between these studies was considerable (*I*^2^ = 66%, *P*=0.02). The meta-analysis using the random-effects model showed that the resting heart rate of the experimental group was significantly better than that of the control group (MD = −4.50, 95% CI: −8.85 to −0.14, *Z* = 2.02, *P*=0.04) (Figure 4). The sensitivity analysis showed that the study by Malmo et al. [15] was the cause of the heterogeneity of the results, but the analysis results did not change, indicating that the results were stable, as shown in Table 5.

#### 3.4.4. Left Ventricular Ejection Fraction (LVEF)

**Figure 5:**
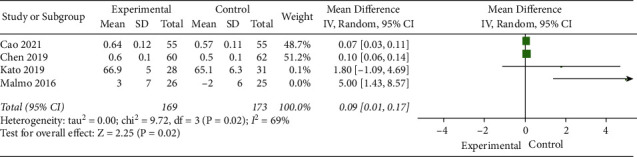
Meta-analysis of the comparison of left ventricular ejection fraction between the two groups.

Four studies [13–15, 17] evaluated the effect of exercise on the LVEF of AF patients. The random-effects model was selected for meta-analysis (*I*^2^ = 69%, *P*=0.02). The results showed that LVEF in the experimental group was significantly better than that of the control group (MD = 0.09, 95% CI: 0.01 to 0.17, *Z* = 2.25, *P*=0.02) (Figure 5). The sensitivity analysis showed that the study by Malmo et al. [15] was the cause of the heterogeneity of the results, but the analysis results did not change, indicating that the results were stable, as shown in Table 6.

#### 3.4.5. Changes in the Quality of Life

**Figure 6:**
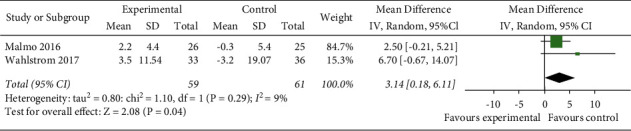
Meta-analysis of the comparison of physical component summary of the two groups.

**Figure 7:**
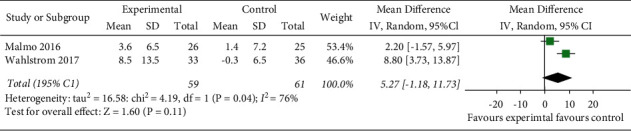
Meta-analysis of the comparison of mental component summary of the two groups.

Two RCTs [12, 15] used SF-36 to evaluate the improvement in quality of life in AF patients with exercise. The random-effects model was selected for the meta-analysis. (*I*^2^ = 9%, *P*=0.29). The meta-analysis showed that the physical condition of the experimental group was better than that of the control group, and the difference was statistically significant (MD = 3.14, 95% CI: 0.18 to 6.11, *Z* = 2.08, *P*=0.04) (Figure 6). The random-effects model was selected, and the improvement of MCS in the experimental group was lower than that of the control group. The difference was not statistically significant (MD = 5.27, 95% CI: −1.18 to 11.73, *P*=0.11) (Figure 7).

## 4. Discussion

RFA is an internationally recognized treatment method to prevent the onset of AF and can effectively maintain normal sinus rhythm for paroxysmal AF and persistent AF. Its primary benefit is to reduce the symptoms related to arrhythmia and improve the quality of life [20–22]. However, studies have found that several risk factors can affect the outcome of RFA, including the type of AF, duration, and complications (such as hypertension, obesity, metabolic syndrome, and sleep apnea syndrome). Currently, cardiac rehabilitation, which focuses on exercise rehabilitation, has been proven to play a positive role in controlling disease risk factors such as coronary heart disease, heart failure, acute myocardial infarction after PCI, and heart transplantation [23, 24]. However, few reports are available on patients receiving exercise rehabilitation after RFA, and the rehabilitation effect is still unknown.

This study explored the effect of exercise on patients with AF after RFA by meta-analysis. The results showed that compared with the control group, planned exercise rehabilitation could significantly improve the exercise tolerance, cardiac function, and physical condition of patients after RFA. The reason was that exercise rehabilitation could improve vascular endothelial function, be anti-inflammatory, reduce myocardial remodeling, and improve myocardial ischemia and antithrombotic effects, thereby effectively reducing the risk factors of AF [25].

Exercise intervention can significantly improve the exercise tolerance level of AF patients after RFA. This study carried out a quantitative analysis of 6MWD and peak VO_2_ indicators, which were the best indicators for evaluating exercise tolerance. The study results were consistent with Morseth et al. [26], who proposed that aerobic exercise could improve the exercise endurance of patients and the physical condition of patients with heart disease after surgery. The report of the postphysical condition is consistent with the conclusion. The reason might be related to the improvement of cardiac output, the increase of peripheral skeletal muscle oxygen utilization, and the correlation between 6MWD and oxygen uptake, thereby improving the exercise tolerance of patients after surgery.

Exercise can improve the heart function of AF patients after RFA. LVEF is one of the indicators reflecting the systolic function of the heart. Regular exercise can increase myocardial glucose uptake, promote the utilization of myocardial substrates, increase cardiac output, and improve left ventricular remodeling. This is especially important for AF patients, who can benefit from the improved systolic function. It is consistent with the effect of exercise intervention in the study by Yagishita et al. [27]. Resting heart rate is a relatively simple and direct indicator of understanding heart function. This study showed that AF patients receiving RFA had their resting heart rate decreased after exercise. The reason might be that exercise inhibited the vagus nerve of the heart activity and the sympathetic nerve activity was weakened [28], which improved the cardiac neuromodulation mechanism. Meanwhile, exercise caused adaptive changes in the structure of myocardial fibers, increased contractility, and strengthened blood supply, thereby reducing the burden on the heart.

Exercise can improve the physical condition of AF patients after RFA. In this study, PCS and MCS were used to reflect the quality of life of patients. After exercise, the physical condition of the patients improved, but their mental condition did not. Relevant studies have confirmed that exercise improves physical condition, but there are controversies about its role in mental health. Nourmohammadi et al. [29] reported that compared with the experimental group, the control group had higher mental health and euphoria levels, while Reed et al. [30] believed that after exercise rehabilitation, patients' mental health levels improved. The reason might be related to the severity of AF and the mode of exercise intervention, such as physical therapist-ledexercise-based cardiac rehabilitation (PT-X), prescription physical activity (PAP) [31], and whether the selection of exercise type was based on the patients' wishes. This indicates that in improving the quality of life of AF patients after RFA, medical staff should increase the initiative of patients, allow them to participate in the formulation of exercise programs, and pay attention to the subjective experience of patients during exercise to promote the mental and physical health of patients.

Due to individual differences among patients with AF, such as age restriction, gender difference, and multiple diseases, the selection of exercise patterns and exercise intensity for patients should be personalized. It is recommended to conduct an individualized pre-exercise evaluation before prescribing exercise to identify whether the patient has high-risk groups with an increased risk of exercise-related sudden death and other cardiovascular events. An RCT study showed that low-intensity and high-intensity exercise increased the exercise capacity of cardiovascular patients by 16% and 14%, respectively [19], thereby reducing the symptoms and burden of AF. Studies have confirmed that the comprehensive care of AF by the ABC pathway plays a potential role in reducing the risk of major adverse consequences [32]. Therefore, some researchers suggest that exercise rehabilitation and the ABC approach can be used together to highlight the advantages of each and maximize the benefits of postoperative prognosis and rehabilitation in patients with AF.

This study has several limitations. First, due to the inconsistency of various exercise schemes included in the study, we cannot further explore the impact of different motion characteristics on the results through subgroup analysis. Secondly, the heterogeneity of AF and the different severity of symptoms among different AF subtypes lead to large population differences and make it difficult to measure the burden of AF. Because the full text of the eight studies was not available, some important evidence may have been omitted. The included literature lacks sufficient evidence to determine the impact of exercise rehabilitation on the risk of postoperative death or hospitalization of AF. It is recommended to strengthen the research on postoperative complications of AF in the later stages. In the future, long-termfollow-ups will be conducted with standard exercise intervention programs, as well as conducting large-scale experimental studies on single and multiple exercise strategies to understand whether exercise intervention has a long-term impact on the indicators in this study and other important clinical results and to provide an effective clinical basis for the promotion of exercise intervention for AF patients after RFA.

To sum it up, the controversy on the role of exercise for AF patients after RFA mainly focuses on whether high-intensity exercise will lead to a poor prognosis, and the effects of mild- and medium-intensity exercise on prevention and treatment have been confirmed [33]; i.e., exercise intervention has positive significance for exercise tolerance, cardiac function, and physical condition of patients with AF. In the future, the formulation of exercise programs should be based on full evaluation, follow the principles of individualization and structuralization, and the decision should be made by both doctors and patients so that the form of exercise, exercise intensity, and frequency of exercise can meet the actual needs of patients, thereby improving patients' participation in sports, helping them maintain long-term exercise, and achieving the purpose of improving the prognosis of patients.

## Figures and Tables

**Table 1:** Basic characteristics of the included studies.

Included studies	Country	Age		Sample size		Intervention measures		Intervention frequency	Intervention period	Outcome indicators
		Experimental group	Control group	Experimental group	Control group	Experimental group	Control group			
Osbak et al. [10]	Denmark	69.5 ± 7.3	70.9 ± 8.3	24	23	Cycling, walking on stairs, running, balling, and interval training	Habitual physical activity	3 times/w, 30 min/each time	12 w	①④
Lakkireddy et al. [11]	USA	60.6 ± 11.5	63.9 ± 7.6	49	53	Yoga training combined with medication	Routine care measures	2 times/w; 60 min/times	12 w	④
Wahlstrom et al. [12]	Sweden	64 ± 7	63 ± 8	33	36	Yoga training combined with medication	Routine drug treatment	1 time/w; 60 min/times	12 w	④⑤
Cao et al. [13]	China	50.9 ± 6.23	51.08 ± 5.81	55	55	Aerobic exercise, mainly walking	Routine interventions	3 times/w	1 y	①③
Chen et al. [14] 2019	China	51.2 ± 7.4	50.6 ± 7.6	60	62	Aerobic exercise, mainly walking	Routine medication and health education	3-4 times/w, performed every other day	1 y	①③
Malmo et al. [15]	Norway	56 ± 8	62 ± 9	26	25	Aerobic exercise, mainly walking/running	Routine habitual exercise	3 times/w; 45 min/	12 w	②③④⑤
Risom et al. [16]	Denmark	60 ± 9	59 ± 12.25	105	105	Sports training and psychological education and consultation	Routine care measures	3 times/w	12 w	1
Kato et al. [17]	Japan	67 ± 10	65 ± 8	28	31	Moderate-intensity endurance and resistance training	Routine care measures	3 times/w; 30 min/times	6 m	①②③④
Joensen et al. [18]	Denmark	62.2 ± 10	60.2 ± 8.9	28	24	Interval training combined with rehabilitation education	Routine treatment	2 times/w; 1 h/time	12 w	①②
Skielboe et al. [19]	Denmark	61.4 ± 3	63.8 ± 3.3	37	33	High-intensity exercise	Traditional low-intensity exercise	2 times/w; 60 min/time	12 w	1

Note: 6MWD: 6-minute walking distance; peak VO_2_: peak oxygen uptake; LVEF: left ventricular ejection fraction; quality of life: physical component summary (PCS) and mental component summary (MCS).

**Table 2:** Quality assessment of the included studies.

Included studies	Randomization methods	Allocation concealment	Blinding		Completeness of outcome data	Selective reporting	Other sources of bias
			Participants and personnel	Assessment			
Osbak et al. [10]	Unclear	Unclear	Yes	Unclear	Complete	No	Unclear
Lakkireddy et al. [11]	Dice rolling	Yes	Unclear	Unclear	Complete	No	Unclear
Wahlstrom et al. [12]	Dice rolling	Yes	Yes	Yes	Complete	No	Unclear
Cao et al. [13]	Number method	Unclear	Unclear	Unclear	Complete	No	Unclear
Chen et al. [14]	Unclear	Unclear	Unclear	Unclear	Complete	No	Unclear
Malmo et al. [15]	Computer	Unclear	No	Yes	Complete	No	Unclear
Risom et al. [16]	Computer	Yes	Yes	Unclear	Incomplete	No	Unclear
Kato et al. [17]	Computer	Yes	No	Unclear	Complete	No	Unclear
Joensen et al. [18]	Number method	No	No	Yes	Complete	No	Unclear
Skielboe et al. [19]	Computer	Unclear	Unclear	Unclear	Complete	No	Unclear

**Table 3:** Sensitivity analysis of the effect of exercise on the 6MWD of AF patients.

Included studies	Heterogeneity test		Overall effect
	*I * ^2^ (%)	*P*	MD (95% CI)
Original meta-analysis	66	0.02	34.42 [3.20 to 65.63]
(Deleted) Joensen et al. [18]	73	0.01	40.81 [3.40 to 78.23]
(Deleted) Kato et al. [17]	75	0.008	35.96 [−3.31 to 75.22]
(Deleted) Osbak et al. [10]	0	0.80	21.53 [5.48 to 37.59]
(Deleted) Cao et al. [13]	74	0.01	38.94 [−8.55 to 86.43]
(Deleted) Chen et al. [14]	72	0.01	41.92 [2.91 to 80.92]

**Table 4:** Sensitivity analysis of the effect of exercise on the peak VO_2_ of AF patients.

Included studies	Heterogeneity test		Overall effect
	*I * ^2^ (%)	*P*	MD (95% CI)
Original meta-analysis	56	0.06	1.52 [0.17 to 2.86]
(Deleted) Joensen et al. [18]	66	0.03	1.61 [0.13 to 3.09]
(Deleted) Kato et al. [17]	63	0.07	1.36 [−0.38 to 3.10]
(Deleted) Malmo et al. [15]	37	0.19	1.05 [−0.19 to 2.29]
(Deleted) Risom et al. [16]	67	0.03	1.42 [−0.68 to 3.51]
(Deleted) Skielboe et al. [19]	12	0.33	2.01 [1.07 to 2.96]

**Table 5:** Sensitivity analysis of the effect of exercise on resting heart rate of AF patients.

Included studies	Heterogeneity test		Overall effect
	*I * ^2^ (%)	*P*	MD (95% CI)
Original meta-analysis	66	0.02	−4.50 [−8.85 to −0.14]
(Deleted) Kato et al. [17]	74	0.009	−5.48 [−11.63 to 0.67]
(Deleted) Lakkireddy et al. [11]	71	0.02	−4.77 [−11.18 to 1.64]
(Deleted) Malmo et al. [15]	40	0.17	−5.92 [−9.63 to −2.21]
(Deleted) Osbak et al. [10]	63	0.04	−3.52 [−7.44 to 0.41]
(Deleted) Wahlstrom et al. [12]	70	0.02	−3.62 [−8.50 to 1.26]

**Table 6:** Sensitivity analysis of the effect of exercise on the left ventricular ejection fraction of AF patients.

Included studies	Heterogeneity test		Overall effect
	*I * ^2^ (%)	*P*	MD (95% CI)
Original meta-analysis	69	0.02	0.09 [0.01 to 0.17]
(Deleted) Kato et al. [17]	76	0.02	0.09 [0.01 to 0.16]
(Deleted) Malmo et al. [15]	19	0.29	0.09 [0.05 to 0.12]
(Deleted) Cao et al. [13]	77	0.01	1.85 [−0.88 to 4.57]
(Deleted) Chen et al. [14]	77	0.01	1.84 [−0.91 to 4.59]

## Data Availability

All data are included within the article.
